# Sun Protection Behavior in Danish Outdoor Workers Following a Multicomponent Intervention

**DOI:** 10.3389/fpubh.2022.885950

**Published:** 2022-04-28

**Authors:** Marie Munk Jakobsen, Ole Steen Mortensen, Kasper Grandahl

**Affiliations:** ^1^The Department of Occupational and Social Medicine, Holbæk Hospital, Copenhagen University Holbæk, Holbæk, Denmark; ^2^Department of Public Health, Section of Social Medicine, University of Copenhagen, Copenhagen, Denmark

**Keywords:** outdoor worker, occupational, sun protection behavior, skin cancer, intervention—behavioral, risk awareness, Danish

## Abstract

**Background:**

Outdoor workers can be exposed to relatively high levels of ultraviolet radiation and are at risk of developing occupational skin cancer. Implementing the use of sun protection in outdoor workers at work is therefore important. The objective of this follow-up study was to evaluate the effect of a multicomponent intervention to improve the use of sun protection in Danish outdoor workers.

**Method:**

A total of 237 Danish outdoor workers were asked to complete surveys in 2016/17 and in 2020. Multicomponent interventions, between surveys, included information on skin cancer risk and use of sun protection, personal dosimetry and skin examination for signs of photodamage and skin cancer. Survey items on awareness of occupational skin cancer risk and perceived importance of sun protection as well as availability and use of sun protection at work were compared and analyzed in relation to the multicomponent intervention.

**Results:**

Overall, the use of sun protection at work increased significantly (composite score [95% CI] 4.0 [3.7, 4.3] in 2016/17 and 4.6 [4.3, 4.9] in 2020, *p* < 0.001). Sunscreen was by far the biggest contributor, and the only type of sun protection used at work, which changed significantly (often/always use 37% in 2016/17 and 52% in 2020, *p* < 0.001). The biggest influence on the increased use of sun protection at work seemed to be a significant increase in the awareness of occupational skin cancer risk (moderate/high 43% in 2016/17 and 63% in 2020, *p* < 0.001) and perceived importance of sun protection at work (moderate/high 69% in 2016/17 and 83% in 2020, *p* < 0.001).

**Conclusion:**

The results of this study indicate that awareness of occupational skin cancer risk as well as the perceived importance and use of sun protection at work in Danish outdoor workers may be improved by means of multicomponent intervention.

## Introduction

Ultraviolet radiation is classified as a group 1 carcinogen by the international Agency for Research on Cancer, and is the main risk factor for developing skin cancer (1–3). Worldwide, the incidence of skin cancer has increased significantly in recent decades (4) warranting an increased focus on preventing solar ultraviolet radiation exposure.

Outdoor workers, in particular, can be exposed to relatively high levels of solar ultraviolet radiation and may thus be at increased risk of developing skin cancer. In Denmark, there are about 400 000 outdoor workers (5), and recent measurements in Danish workers have shown levels of exposure to ultraviolet radiation in outdoor workers that are approximately four times higher than that of indoor workers (6). Outdoor workers in several other European countries are similarly exposed to relatively high levels of occupational solar ultraviolet radiation (7, 8).

In two systematic reviews from 2011, outdoor workers were shown to have a significantly higher risk of developing keratinocyte cancer compared to non-exposed workers (9, 10).

Sun safety at work can be improved by the use of sun protection such as: avoiding the sun during midday, sunscreen, long sleeved shirt and trousers and a wide brimmed hat (11). In 2019, The Danish Working Environment Authority issued a news item recommending the use of sun protection at work, which in 2021 became a requirement to make sunscreen available in outdoor workplaces (12). The primary recommendation was issued one year after the publication of a survey study that showed limited awareness of occupational skin cancer risk, perceived importance and use of sun protection at work in Danish outdoor workers (13). It is unclear if these recommendations and requirements have had any impact.

Several sun safety campaigns have tried to encourage more and better use of sun protection in the general population (14). However, studies have shown that sun safety campaigns only have a short term effect, unless they are repeated and supplemented with education, policy, and environmental strategies (15). A German study showed that a 16-year period of repeated sun safety campaigns reduced the amount of sun burns in the general population from 25.9 to 17.5% (14).

Some studies have researched the effect of workplace sun protection policies and measures, but with inconclusive results (16, 17). This includes workplace education and knowledge about skin cancer, both of which have showed mixed results (16). In a study of Australian outdoor workers, education combined with skin examination to modify health risk behavior and reduce skin cancer risk was found to improve sun protection behavior (18). Attitude towards sun protection is also believed to affect sun protection behavior. In a systematic review from 2012, including 16 multicomponent intervention studies, 13 studies found a positively increased sun protection behavior, and eight studies measured a change in attitude towards skin cancer, of which only one study found a positive short time effect in outdoor workers (19).

The best way to improve sun protection behavior at work is probably by multicomponent intervention including sun safety policy, structural changes, education, skin examination, and role models (20, 21). This was shown in a study among Israeli outdoor workers, using multicomponent intervention including repeated skin examination, education, clinical training, and availability of personal sun protection gear, with consequent significantly improved sun protection behavior at work. A high proportion (80%) of the Israeli outdoor workers sustained this behavior one year after the intervention (20). The same was observed in an intervention study from Queensland, where the use of multicomponent intervention including sun safety policy at work, structural and environment changes towards sun protection, personal protective equipment, education and awareness, role modeling and skin examination led to increased use of sun protection in outdoor workers (21).

Previous studies indicate that single-component interventions are not enough to change sun protection behavior in outdoor workers. The effects of multicomponent intervention on sun protection behavior have not previously been studied in Danish outdoor workers. The aim of this study was to evaluate the sun protection behavior of Danish outdoor workers as a four-year after their participation in a PhD project that included multicomponent intervention, and one year after a recommendation on the use of sun protection at work by the Danish Working Environment Authority.

## Method

A follow-up study of changes in the sun protection behavior of Danish outdoor workers after a four-year period and multiple interventions aimed to prevent exposure to ultraviolet radiation and the development of skin cancer, as part of the PhD project “*Solar ultraviolet radiation exposure, sun protection behavior and skin photodamage in Danish Workers”* (5). Recruitment was originally carried out in 2016/17 by means of convenience sampling among a large number of Danish companies, municipalities and unions. Participants had to be active in the labor market (inclusion criteria) and could not have insufficient Danish language skills (exclusion criteria, 13). In 2016/17, 499 participants completed the PhD study questionnaire including items on demographic characteristics, occupational history, awareness of skin cancer risk and use of sun protection at work, at leisure, and on sun holiday (13). In 2020, the same participants were contacted by email. In case of no response, they received a text-message or a telephone call and asked to complete a shortened follow-up version of the PhD study questionnaire, including the exact same items in terms of awareness of occupational skin cancer risk, perceived importance and use of sun protection at work (13) (Supplementary Material). The PhD study questionnaire included 47 items, of which 33 were reused in the follow-up study questionnaire. Most of the items were new constructs developed particular for the PhD study. Before use, three experienced researchers reviewed and six representative workers completed and evaluated the PhD study questionnaire to improve its face validity (13). Between survey interventions included: two-weeks personal ultraviolet radiation dosimetry between May 2016 and May 2017, a skin examination for signs of photodamage and skin cancer in late 2016, and one-time written feedback to participants on personal exposure to solar ultraviolet radiation, skin cancer risk and recommendations on the use of sun protection in 2017 (5). None of the interventions were linked to the Danish Working Environment Authority recommendations regarding the use of sun protection at work. In this study, multicomponent intervention is defined as an intervention with at least two components.

In this study, participants that predominantly work outside or work equal parts outdoor and indoor were categorized as outdoor workers. This choice was based on results from a recent Danish dosimetry study which showed that workers who work outdoors half the time are exposed above the International Commission on Non-Ionizing Radiation Protection threshold value for ultraviolet radiation exposure of 1.0–1.3 SED per 8-hour work period (6). Also, for each outdoor worker, as a measurement of overall use of sun protection, a composite score (0–12 points) was calculated based on their answer (never = 0, rarely = 1, often = 2, always = 3) for each of the four sun protection items (avoid sun during midday, long trouser and sleeves, wide brimmed hat and sunscreen).

### Statistical Analysis

McNemar's test was used to test for differences in awareness of occupational skin cancer risk as well as perceived importance, availability and use of sun protection at work between 2016/17 and 2020. Chi2- and *t*-test with standard deviation and p-values were used as statistics. All participants in the analysis completed the survey in both 2016/17 and in 2020. We did a further analysis to assess if statistically significant changes in the use of sun protection at work were related to skin examination, awareness of occupational skin cancer risk, or perceived importance and availability of sun protection at work. Sensitivity analyzes were done for participants with multicomponent intervention. Multiple variate regression was done to assess the influence of demographic variables on change in composite score. Statistical significance was determined using α = 0.01. The JMP 14 statistics program was used.

## Results

**Figure 1:**
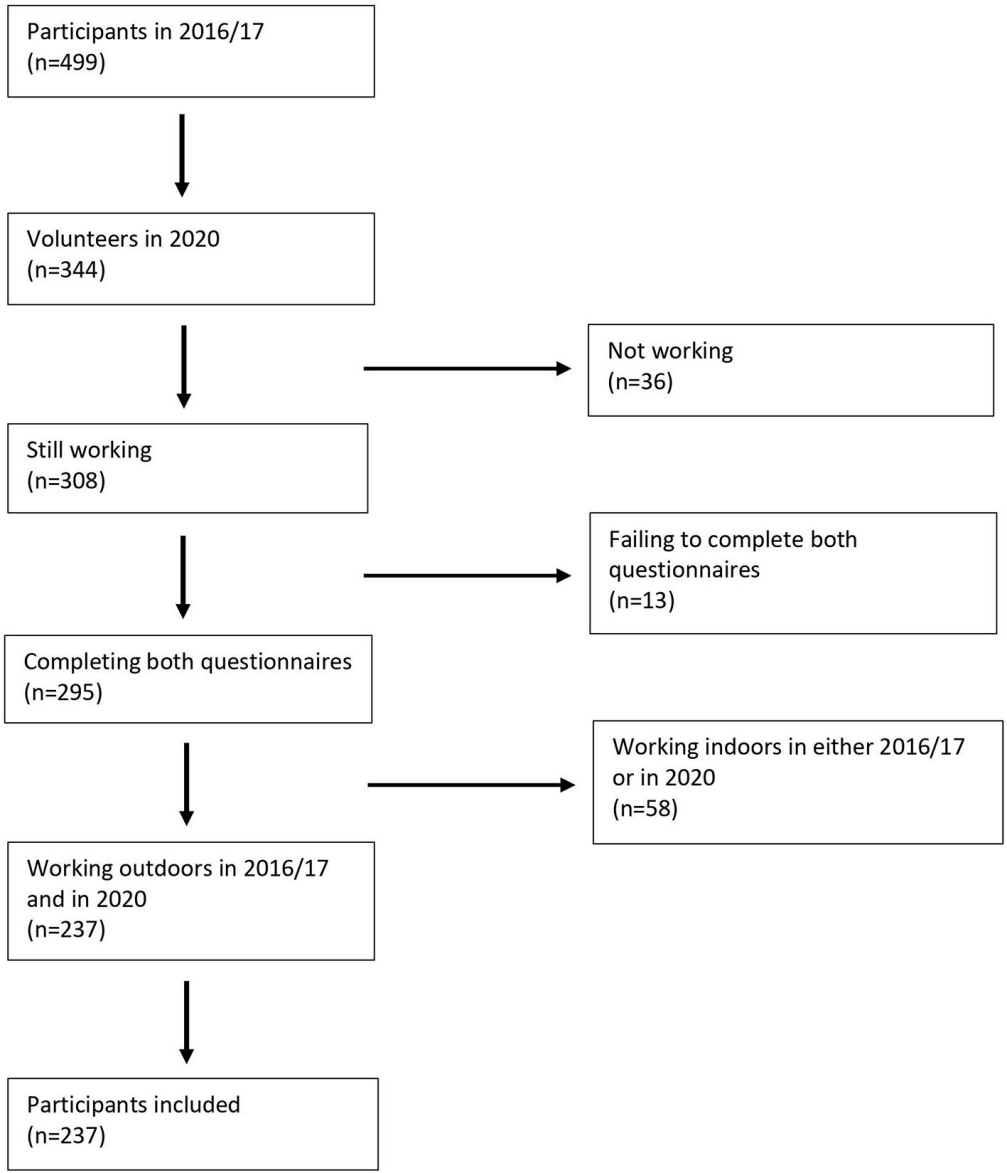
Flowchart.

Of the original 499 participants, 344 agreed to complete the follow-up study questionnaire. Hereof, 308 were still working. Of these, ten did not sufficiently complete the original PhD study questionnaire and three did not complete the follow-up study questionnaire. Of the remaining 295 volunteers, 58 were excluded since they were working indoor either in 2016/17 or in 2020, resulting in a final tally of 237 outdoor workers without job changes completing both study questionnaires, as participants in this study. Figure 1 shows the process in a flowchart.

Table 1 shows the baseline characteristics of the participants based on their responses in the original and in the follow-up study questionnaire. Most participants were men (77%) with a mean age of 45.3 years in 2016/17. The main part of participants had elementary and vocational school as their highest level of education (68%). The profession with most participants was gardener, followed by carpenter, roofer, postal worker, dockworker, road workers and others. Most had skin type 3, no history of skin cancer, never been smokers and drank/consumed less than 10 units of alcohol a week. All participants received information and education on skin cancer risk and use of sun protection and all but six participants performed personal dosimetry. About half of the participants (*n* = 129) had a skin examination done for signs of photodamage and skin cancer. In total, 231 (97%) of all participants had multicomponent intervention.

**Table 1:** Baseline characteristics of the participants.

	**Responses**	**Results (*N* = 237)**
Sex^a^	Male	183 (77%)
	Women	54 (23%)
Age^a^	Mean (Std. Dev)	45.3 (10.3)
Educational level^a^	Elementary or vocational school	161 (68%)
	High school	19 (8%)
	Higher education	46 (19%)
	Other	11 (5%)
Profession^a^	Gardener	58 (24%)
	Carpenter	20 (8%)
	Roofer	22 (9%)
	Postal worker	17 (7%)
	Dock worker	15 (6%)
	Road worker	12 (5%)
	Others^f^	93 (41%)
Skin type^a^, ^d^	Type 1	5 (2%)
	Type 2	61 (26%)
	Type 3	103 (43%)
	Type 4	56 (24%)
	Type 5	12 (5%)
Personal history of skin or lip cancer^b^	Yes	12 (5%)
	No	225 (95%)
Smoking^b^	Never	168 (71%)
	Former	28 (12%)
	Current	41 (17%)
Alcohol^b^	Never	45 (19%)
	Less than 10units/w	166 (70%)
	More than 10units/w	26 (11%)
Skin examination^c^	Yes	129 (54%)
	No	108 (46%)
Dosimetry^e^	Yes	231 (97%)
	No	6 (3%)
Employed in same job^b^	Yes	206 (87%)
	No	31 (13%)
Working outdoor or equal indoor/outdoor^b^	Outdoor	187 (79%)
	Equal indoor/ outdoor	50 (21%)

a *Results from 2016/17*.

b *Results from 2020*.

c *In study late 2016*.

d *According to the Fitzpatrick scale (25)*.

e *In study between May 2016 and May 2017*.

f *Concrete technician, surveyor, machine operator/driver, mason, porter, renovation worker, scaffolding worker, road worker, sewer construction worker, mason, various outdoor workers*.

Table 2 compares the participants' answers in the PhD study questionnaire and the follow-up study questionnaire as to awareness of occupational skin cancer risk, perceived importance, availability and use of sun protection at work. The table shows that awareness of occupational skin cancer risk has changed significantly (*p* < 0.001) towards a higher incidence of moderate-high awareness of occupational skin cancer risk in 2020 (63%) compared to in 2016/17 (43%). Perceived importance of sun protection at work has similarly changed significantly (*p* < 0.001) towards a higher incidence of moderate-high perceived importance of sun protection at work in 2016/17 (69%) compared to 2020 (83%).

**Table 2:** Comparison of the participants' awareness of sun safety, availability of sun protection, and use of sun protection at work in 2016/17 and 2020, respectively.

		**2016/17**	**2020**	**Chi^**2**^/ t-test**	***p***
**Awareness of sun safety at work (*****N*** **=** **237)**					
Awareness of occupational skin cancer risk	Not considering	110 (46%)	71 (30%)	35.87	<0.001
	No or low	26 (11%)	16 (7%)		
	Moderate	56 (24%)	88 (37%)		
	High	45 (19%)	62 (26%)		
Perceived importance of sun protection at work	No	27 (11%)	9 (4%)	29.74	<0.001
	Low	47 (20%)	30 (13%)		
	Moderate	88 (37%)	103 (43%)		
	High	75 (32%)	95 (40%)		
**Workplace availability of sun protection (*****N*** **=** **237)**					
Avoid the sun during midday	Yes	14 (6%)	30 (13%)	9.85	0.002
	No	223 (94%)	207 (87%)		
Long trouser and sleeves	Yes	219 (92%)	204 (86%)	10.76	0.013
	No	16 (7%)	33 (14%)		
	Missing	2 (1%)	-		
Wide brimmed hat	Yes	111 (47%)	129 (54%)	5.19	0.159
	No	125 (53%)	108 (46%)		
	Missing	1 (1%)	-		
Sunscreen	Yes	70 (30%)	126 (53%)	35.66	<0.001
	No	167 (70%)	111 (47%)		
**Use of sun protection at work (*****N*** **=** **235)**					
Composition score	Mean [95% CI]	4.0 [3.7, 4.3]	4.6 [4.3, 4.9]	t_(235)_ = 5.32	<0.001
	Std. Dev	2.0	2.1		
Long trouser and long sleeves	Never	22 (9.5%)	30 (13%)	8.48	0.205
	Rare	118 (50%)	115 (49%)		
	Often	87 (37%)	73 (31%)		
	Always	8 (3.5%)	17 (7%)		
Wide brimmed hat	Never	113 (48%)	93 (40%)	17.48	0.008
	Rare	58 (25%)	64 (27%)		
	Often	37 (16%)	47 (20%)		
	Always	27 (11%)	31 (13%)		
Sunscreen	Never	51 (21%)	34 (14.5%)	33.18	<0.001
	Rare	99 (42%)	78 (33.5%)		
	Often	65 (28%)	85 (36%)		
	Always	20 (9%)	38 (16%)		
Avoid the sun during midday	Never	127 (54%)	102 (43%)	20.44	0.002
	Rare	91 (39%)	105 (45%)		
	Often	15 (6%)	25 (11%)		
	Always	2 (1%)	3 (1%)		

*Analysis done for participants answering both questionnaires with a few missing values*.

With regard to availability of sun protection in the workplace, a significant difference was found for the use of sunscreen (*p* < 0.001) and avoiding the sun during midday (*p* = 0.002) at work between 2016/17 and 2020. As to the use of sun protection at work, a significant increase in composite score (*p* < 0.001) was shown between 2016/17 and 2020. More importantly, a significant difference was found in the use of sunscreen (*p* < 0.001), avoiding sun during midday (*p* = 0.002) and wearing a wide-brimmed hat (*p* = 0.008) at work between 2016/17 and 2020. Hereof, the percentage change was by far the largest for sunscreen at work, used often or always by 37% in 2016/17 and by 52% in 2020.

Sensitivity analyzes including only participants who had received multicomponent intervention did not change the significance of the results.

Table 3 shows the use of sunscreen at work in relation to skin examination, availability of sunscreen, awareness of occupational skin cancer risk and perceived importance of sun protection at work. A significant association was found between use of sunscreen at work and both awareness of occupational skin cancer risk (*p* < 0.001) and perceived importance of sun protection (*p* < 0.001) at work. By looking at the percentage differences, it seems likely that an increase towards a higher awareness of occupational skin cancer risk and perceived importance of sun protection increased the use of sunscreen at work. The table also shows that the use of sunscreen at work was not significantly related to neither skin examination nor availability of sunscreen in the workplace in 2020.

**Table 3:** Participants' use of sunscreen in relation to skin examination, workplace availability of sunscreen, awareness of occupational skin cancer risk and perceived importance of sun protection at work in 2020.

**Skin examination (*****N*** **=** **235)**						
**Use of sunscreen**	**Yes**		**No**		**Chi** ^**2**^	***p***
Never	17 (13%)		17 (16%)		2.04	0.565
Rare	39 (30%)		39 (36%)			
Often	52 (41%)		33 (31%)			
Always	20 (16%)		18 (17%)			
**Workplace availability of sunscreen (*****N*** **=** **236)**						
**Use of sunscreen**	**Yes**		**No**		**Chi** ^**2**^	***p***
Never	16 (13%)		18 (16%)		1.23	0.772
Rare	40 (32%)		38 (35%)			
Often	49 (39%)		36 (33%)			
Always	20 (16%)		18 (16%)			
**Awareness of occupational skin cancer risk (*****N*** **=** **236)**						
**Use of sunscreen**	**Not considering**	**No or low**	**Moderate**	**High**	**Chi** ^**2**^	***p***
Never	26 (37%)	4 (25%)	4 (5%)	1 (2%)	76.60	<0.001
Rare	30 (42%)	8 (50%)	28 (16%)	12 (19%)		
Often	12 (17%)	3 (19%)	40 (46%)	29 (47%)		
Always	3 (4%)	1 (6%)	14 (32%)	20 (32%)		
**Perceived importance of sun protection at work (*****N*** **=** **237)**						
**Use of sunscreen**	**No**	**Low**	**Moderate**	**High**	**Chi** ^**2**^	**p**
Never	5 (56%)	10 (33,3%)	18 (18%)	1 (1%)	91.40	<0.001
Rare	-	13 (43,3%)	46 (45%)	16 (17%)		
Often	3 (33%)	6 (20%)	34 (33%)	46 (48%)		
Always	1 (11%)	1 (3,3%)	4 (4%)	32 (34%)		

A similar analysis was made/done for avoiding the sun around midday and use of a wide brimmed hat. In this, a statistical significant association was found only between workplace availability and use of avoiding the sun around midday (*p* < 0.001). However, the numbers were quite small with only ten outdoor workers having both availability and always or often use of avoiding the sun around midday in 2020.

In addition, to see whether the change in the composite score could be explained by differences in demographic variables in 2016, we performed a multiple linear regression with change from 2016/17–2020 in the composite score as the dependent variable and sex, age, skin type, and work and educational level as explanatory variables. The result of the multiple linear regression model showed that the observed change in composite score could not be explained by differences in the dependent variables *F*_(15, 219)_ = 1.27, *p* = 0.221.

## Discussion

The results show a significant increase in the awareness of occupational skin cancer risk, perceived importance of sun protection and use of sun protection at work in Danish outdoor workers, following a four-year period, including multicomponent intervention aimed to prevent exposure to ultraviolet radiation and the development of skin cancer.

The modest increase in composite score for overall use of sun protection appears to be primarily driven by a marked increase in use of sunscreen at work. This more so than both avoiding the sun around midday and wearing a wide-brimmed hat at work. The increased use of sunscreen was, somewhat surprisingly, unrelated to the increased availability of sunscreen at work. Moreover, neither personal dosimetry nor skin examination alone lead to changes in the use of sunscreen at work. Thus, indicating a possible combined effect of personal dosimetry, skin examination as well as information on skin cancer risk and recommendations on use of sun protection to explain the increased use of sun protection, mainly sunscreen, in the workplace.

The results of this study thus seem to support the notion that the best way to improve sun protection at work, especially the use of sunscreen, in Danish outdoor workers is through the effects of multicomponent intervention. This finding is in line with international studies demonstrating the efficacy of multicomponent interventions to increase the use of sun protection at work, in particular with regard to personal protective equipment such as sunscreen (15, 22).

The marked increase in Danish outdoor workers' use of sunscreen at work may be due to it being more readily available and well known compared to other types of sun protection. Also, outdoor workers are likely to have a significantly higher impact on the use of sunscreen compared to other types of sun protection during working hours. Whatever the reason, the increased use of sunscreen by Danish outdoor workers is a step in the right direction and something to build on in terms of improving sun safety at work. That being said, sunscreen is generally considered the least effective type of sun protection and best used in combination with other more effective types of sun protection (23). It is therefore important to emphasize an additional need for sun protective clothing, and to avoid the sun around midday whenever possible during working hours.

In a recent systematic review, the immediate feedback from personal monitoring of physical activity was shown to effectively increase physical activity in a Danish adult population (24). In this study, although feedback from personal dosimetry of ultraviolet radiation was not immediate, but rather delayed by several months, the increased use of sun protection at work may, to some extent, be attributed to the use of personal dosimetry. However, it is not possible to assess such a correlation, as virtually all outdoor workers participated in this intervention. The potential of using personal dosimeters with immediate feedback to increase sun safety at work is nevertheless an interesting research question that should be investigated further.

The main strength of this study is the use of repeated measures in that the same cohort of outdoor workers were being measured using the same dependent variables with 3–4 years' intervals. Also, the inclusion of a broad selection of professions representing outdoor workers allow for a reasonably wide generalization of results. Although not all participants were subject to multicomponent intervention, complete and unbiased knowledge of single-component interventions for each participant allowed for a detailed and reliable analysis.

The study is limited in terms of investigating a possible confounding effect of the recommendations by the Danish Working Environment Authority regarding the use of sun protection at work by a lack of data. The fact that skin cancer risk perception as well as the use of sun protective measures both increase with age, and the risk that some participants may have engaged in other potential confounding measures, i.e. a second skin examination between surveys, are also potential confounders. The PhD study questionnaire was evaluated only with respect to face-validity and no other important psychometric properties such as reliability and norming or sensitivity to change. Additionally, self-evaluated use of sun protection may lead to over- or underestimation, although this is likely to remain the same for each participant over a four-year period and thus not significantly affect comparisons in this study. Moreover, as in the original study, selection bias and consequent low generalization of results cannot be ruled out. Besides, the use of composite scores may lead to skewed results. Also, the composite score assigns the same weight to each component, suggesting that each is equally effective as sun protection, which has been taken into account in the analysis and discussed in more detail. Lastly, the study is limited by not having a control group and as such, this is not an intervention study.

## Conclusion

Based on the findings in this study, it seems possible to influence the awareness of occupational skin cancer risk and use of sun protection in Danish outdoor workers positively during working hours by multicomponent intervention. Clearly, the greatest effect is seen/observed for the use of sunscreen. However, when it comes to sun protective clothing and avoiding the sun around midday, a higher degree of involvement from the employer in terms of workplace policy and equipment availability and/as well as from the Danish Working Environment Authority in terms of rules and regulations for sun protection at work is needed.

## Data Availability Statement

The raw data supporting the conclusions of this article will be made available by the authors, without undue reservation.

## Ethics Statement

Ethical approval was not provided for this study on human participants because this is in accordance with recommendations by The Zealand Ethical Scientific Committee and Data Monitoring Authority. Written informed consent for participation was not required for this study in accordance with the national legislation and the institutional requirements.

## Author Contributions

KG and OM planned the project. MJ and KG acted as main co-authors. MJ carried out the statistical data analysis. OM supervised the work. All authors has contributed in the final version of the paper.

## Funding

The original study was funded by the Research promoting fund for clinical professors employed in Region Zealand and at the University of Copenhagen, the Else and Mogens Wedell-Wedellsborg Foundation, and the Region Zealand Health Science Research Fund. Grant Award Number: 15-00034.

## Conflict of Interest

The authors declare that the research was conducted in the absence of any commercial or financial relationships that could be construed as a potential conflict of interest.

## Publisher's Note

All claims expressed in this article are solely those of the authors and do not necessarily represent those of their affiliated organizations, or those of the publisher, the editors and the reviewers. Any product that may be evaluated in this article, or claim that may be made by its manufacturer, is not guaranteed or endorsed by the publisher.
