# Supplementary material for: Sun Protection Behavior in Danish Outdoor Workers Following a Multicomponent Intervention
Source: Front Public Health. 2022 Apr 28;10:885950. doi: 10.3389/fpubh.2022.885950 (PMC9098216; doi:10.3389/fpubh.2022.885950)
Supplement: Supplementary file 1 [file Data_Sheet_1.pdf]

Appendix 1

The follow-up study questionnaire in 2020

Are you still working?
-Yes, no

What is your current profession?
-Free text

Where are you employed?
-Free text

Are you employed in the same job as in 2016/17?
Yes, no

What is your status as outdoor or indoor worker?
-Outdoor worker, equal parts outdoor and indoor worker, indoor worker

How many hours per week did you work outside last summer?
-None, few, 10-20, 21-30, 31-40, >41 hours

What is your smoking status?
-Nonsmoker, former smoker, 0-10, 11-20, 21-30, >30 cigarettes a day

How much alcohol do you drink?
-No alcohol, 0-10, 11-20, 21-30, >30 items a week

Have you/ your relatives ever had skin or lip cancer (2 items)?
-Yes, skin cancer, lip cancer, skin and lip cancer, no

Which type of skin cancer of lip cancer have you/ your relatives had earlier (2 items)?
-Basal cell carcinoma, squamous cell carcinoma, malignant melanoma, other type of skin cancer, I do not know

What do you think of the risk of occupational skin cancer?
-The risk is high, moderate, low, insignificant, I do not think about it

Do you think it is important to protect your skin from sunlight during outdoor work in the summer?
-Yes to a high degree, moderate degree, low degree, no

Do your employer provide a wide brimmed hat or cap (not safety helmet)/ long trousers and shirt with sleeves / avoiding the sun around noon /sunscreen as sun protection at work (4 items)?
-Yes, no

Do your employer provide information on prevention of sunlight exposure at work?
-Yes, no

Is your employer aware of protecting his employees from sunlight during outdoor work in the summer?
-Yes, no

Do you use long trousers and shirt with sleeves/ a wide brimmed hat/ sunscreen/ avoid the sun around noon respectively at work/ outdoor leisure/ on sun holiday (12 items)?
-Always, often, rarely, never

Use of long trousers and shirt with sleeves/ a wide brimmed hat/ sunscreen/ avoiding the sun around noon in the summer can reduce my risk of skin cancer?
-Strongly agree, agree, disagree, strongly disagree

Of the following, which is the most significant skin, cancer risk factor in your opinion?
-Sunburn, solarium use, sun holidays, working outdoor, outdoor stay at leisure/ I don't know

May we contact you again if we need more information?
-Yes, no
